# Interleukin 11 is upregulated in uterine lavage and endometrial cancer cells in women with endometrial carcinoma

**DOI:** 10.1186/1477-7827-8-63

**Published:** 2010-06-17

**Authors:** Joanne Yap, Lois A Salamonsen, Tom Jobling, Peter K Nicholls, Evdokia Dimitriadis

**Affiliations:** 1 Prince Henry's Institute of Medical Research, Clayton VIC, 3168, Australia; 2 Department of Obstetrics and Gynaecology, Monash Medical Centre, Clayton, VIC, 3168, Australia

## Abstract

**Background:**

Interleukin (IL) 11 is produced by human endometrium and endometrial cancer tissue. It has roles in endometrial epithelial cell adhesion and trophoblast cell invasion, two important processes in cancer progression. This study aimed to determine the levels of IL11 in uterine lavage fluid in women with endometrial cancer and postmenopausal women. It further aimed to determine the levels of IL11 protein and its signaling molecules in human endometrial cancer of varying grades, and endometrium from postmenopausal women and IL11 signalling mechanisms in endometrial cancer cell lines.

**Methods:**

IL11 levels in uterine lavage were measured by ELISA. IL11, IL11 receptor(R) α, phosphorylated (p) STAT3 and SOCS3 were examined by immunohistochemistry in endometrial carcinomas and in control endometrium from postmenopausal women and normal cycling women. The effect of IL11 on pSTAT3/STAT3 and SOCS3 protein abundance in endometrial cancer cell lines and non-cancer endometrial epithelial cells was determined by Western blot.

**Results:**

IL11 was present in uterine flushings and was significantly higher in women with Grade 1 carcinomas compared to postmenopausal women (p < 0.05). IL11 immunostaining was significantly elevated in the endometrial tumour epithelial cells from Grade 1 and 3 compared to endometrial epithelium from postmenopausal and cycling women. IL11Rα immunostaining intensity was increased in cancer epithelium in the Grades 1 and 2 tumours compared to epithelium from postmenopausal women. Both IL11 and IL11Rα localized to vascular endothelial and smooth muscle cells while IL11 also localized to subsets of leucocytes in the cancer tissues. pSTAT3 was found in both the tumour epithelial and stromal compartments but was maximal in the tumour epithelial cells, while SOCS3 was predominantly found in the tumour epithelial cells. pSTAT3 staining intensity was significantly higher in Grade 1 and 2 tumour epithelial cells compared to epithelial cells from cycling and postmenopausal women. SOCS3 staining intensity did not differ between between each tumour and postmenopausal endometrial epithelium but SOCS3 in cycling endometrium was significantly higher compared to postmonopausal and Tumour Grades 2 and 3. IL11 increased pSTAT3/STAT3 in all tumour cell lines, while SOCS3 abundance was increased only in one tumour cell line.

**Conclusions:**

The present study suggests that IL11 in uterine washings may be useful as a diagnostic marker for early stage endometrial cancer. It indicates that IL11, along with its specific receptor, IL11Rα, and downstream signalling molecules, STAT3 and SOCS3, are likely to play a role in the progression of endometrial carcinoma. The precise role of IL11 in endometrial cancer remains to be elucidated.

## Background

Endometrial cancer is the most common gynaecological malignancy [1]. Since it typically affects postmenopausal women, a significantly increased risk occurs in women from age 40 and thus endometrial cancer is increasingly frequent in many advanced countries [1]. The invasion of endometrial cancer cells through the myometrium and their migration to the nearby lymph nodes are key factors related to its poor prognosis [2]. Despite a relatively high incidence of uterine cancer, particularly in postmenopausal women, a suitable screening test is not available [3]. Additionally, despite advances in the treatment of endometrial cancer, the increasing death rate associated with the disease is increasing demonstrating new treatments are required [4]. Endometrial cancer or adenocarcinoma (type 1), which accounts for about 90% of endometrial cancers, begins in the glandular epithelial cells of the endometrium. Factors that influence endometrial epithelial cell function and are upregulated early in the disease may therefore prove to be critical potential diagnostic and therapeutic targets.

Interleukin (IL) 11 belongs to the IL6 family of cytokines and signals via a heterodimeric complex of IL11 receptor (R) α and gp130. The cellular responses of IL11 are induced by the activation of downstream Janus kinases (JAK) that phosphorylate the latent cytoplasmic transcription factors, signal transducer and activator of transcription (STAT) [5]. Phosphorylated (p) or activated STAT proteins translocate to the nucleus to modulate gene transcription [6]. Cytokine signalling is tightly regulated by a variety of mechanisms [7]. The inducible suppressor of cytokine signalling (SOCS) proteins, a family with 8 members (SOCS1-SOCS7 and CIS), are expressed in response to cytokine stimulation of STAT phosphorylation acting in a negative feedback mechanism to hinder the activities of cytokine receptors [8,9]. IL11 signals via pSTAT3 in human endometrial epithelial cells [10,11] and stimulates SOCS3 in human endometrial cells [9]. IL11 is expressed by endometrial glandular epithelium in women during the menstrual cycle [12]. A recent study had identified that IL11 and IL11Rα are expressed in endometrial cancer [13], although there are no studies comparing the levels of IL11 protein in endometrial cancer and postmenopausal women in whom the vast majority of endometrial cancers develop. It is also not knownswhether IL11 downstream signalling is active in endometrial cancer, which would suggest a role for IL11 in carcinogenesis.

Numerous studies have suggested that IL11 has roles in human gastric, prostate and bone cancer [14-17]. In addition critical roles for pSTAT3 and SOCS3 in cancer have also been proposed [8,18,19]. Tumor cell survival depends on the cells' ability to adhere to, migrate and invade through the tissue and to metastasize into other organs and tissues [20]. We recently showed that IL11 regulates human endometrial epithelial cell adhesion and the migration and invasion of human trophoblast cells [10,21,22]. It has also been suggested that factors present in uterine lavage fluid correlate with the presence of endometrial cancer [23].

In the current study, we determined the levels of IL11 in uterine lavage in women with endometrial cancer and postmenopausal controls. We compared IL11, IL11Rα, pSTAT3 and SOCS3 protein in human endometrial carcinomas of varying histologic grades with endometrium from postmenopausal and cycling women. We determined the effect of IL11 on its downstream signaling molecules in endometrial cancer and non-cancer endometrial epithelial cells.

## Methods

### Patients and tissues

Endometrial cancer tissue biopsies (N = 16) were collected from postmenopausal women undergoing total abdominal hysterectomy for endometrial carcinoma at the Monash Medical Centre Melbourne, Australia. The Human Ethics Committee approved the research project and informed consent was obtained from each patient participating in this study. Details of individual patients are provided in Table 1. All tissues were examined and tumors were graded histologically by a specialist gynecological pathologist according to the guidelines of the International Federation of Gynecology and Obstetrics (FIGO, 1998). In this system the presence of vascular/lymphatic invasion was noted and the depth of myometrial invasion was classified as either: no invasion, < 50% myometrial invasion or > 50% myometrial invasion. Biopsies of endometrium were also obtained from postmenopausal women (N = 4) undergoing minor gynaecological procedures unrelated to endometrial pathology. Histological examination by a specialist gynecological pathologist confirmed whether the endometrium from post-menopausal women was atrophic or active. A large majority of endometrium collected from postmenopausal women are atrophic as they are no longer under hormonal control so that with very little endometrium is present, thus we were very limited in the number of tissue samples of 'active' endometrium we could include in this study. We therefore also collected endometrium from proliferative phase endometrium (N = 10) as a second group of non-tumour normal controls. The control proliferative endometrial biopsies were collected at curettage from women between day 7 and 13 of their menstrual cycle that were scheduled for tubal ligation or were undergoing testing for tubal patency. Tissues were assessed by a pathologist and had no obvious endometrial pathology. The women had no steroid treatment or other medication for at least 2-3 months before the collection of tissue. Written informed consent was obtained from each patient and the study was approved by the Southern Health Human Research and Ethics committee. All endometrial biopsies were fixed overnight in 4% neutral buffered formalin, prior to routine paraffin embedding.

**Table 1:** Clinical characteristics of the patients used in this study

Patient no.	Age	Menopausal status	Cancer Grade	%MI
1	65	Post	1	0
2	56	Post	1	29
3	84	Post	1	80
4	34	UK	1	0
5	78	Post	1	4
6	73	Post	2	38
7	52	UK	2	UK
8	60	Post	2	18
9	88	Post	2	73
10	63	Post	2	100
11	54	Post	3	38
12	59	Post	3	33
13	77	Post	3	25
14	UK	UK	3	UK
15	68	Post	3	13
16	55	UK	3	77

Post: post-menopausal; UK: unknown; % MI: % myometrial invasion.

## Features of patients

For immunohistochemistry studies: There were 16 cancer patients, with an age range of 34-88 years (mean age = 64.4 years with standard deviation = 14.1) (Table 1), while the postmenopausal women had an age range of 51-60 years with a mean age of 54.4 years and a standard deviation of 4.0. Normal cycling women were aged between (29-41). All four of the postmenopausal women had "active" endometrium. Five or six biopsies were collected from each histologic Grades 1, 2 and 3 carcinomas. All patients were diagnosed with endometrioid adenocarcinoma tumors. Myometrial invasion was present in 85.7% of patients; of these, 66.7% had invasion to less than 50% of the myometrium, and 33.3% had invasion to 50% or more of the myometrium (Table 1). The presence of vascular/lymphatic invasion, as assessed by tumor histopathology, was apparent in 56% of patients (data not shown).

Uterine lavage were collected from a subgroup of the women with endometrial carcinoma (Grade 1 N = 4, Grade 2 N = 5, Grade 3 N = 6; mean age = 69.3 years with standard deviation = 12.9) and postmenopausal controls (N = 4, mean age = 73.7 and a standard deviation of 11.1).

### IL11 in uterine fluid

Uterine lavages (uterine washings) were collected from postmenopausal women (N = 4) and women with endometrial cancer above except for women with Grade 1 carcinoma where washings were collected from 4 women instead of 5 women (Grade 1-3 (N = 4; 5; 6 respectively) as previously described [24]. Uterine fluid from all women was concentrated 3-4 fold using Nanosep microconcentration devices with a 3 K cut-off (Pall Life Sciences, East Hills, NY). IL11 was then measured in the samples by ELISA as previously described [25].

### IL-11 and IL-11Rα immunohistochemistry

Immunohistochemistry for IL11 and IL11Rα was performed as described previously [25] using a monoclonal anti-huIL-11 (5E3) and antihuIL-11Rα (4D12) antibodies (generous gifts from Dr. Lorraine Robb). Briefly, paraffin sections (5 μm) were dewaxed in histosol and rehydrated in a graded series of ethanol. Endogenous peroxidase activity was quenched by immersion in 3% H_2_O_2 _in methanol for 10 min. Non-specific staining was blocked using a blocking solution of 10% normal horse serum (Sigma-Aldrich Inc., Missouri, USA) and 2% normal human serum, diluted in 1× Tris-buffered saline (TBS) for 30 min. Primary antibodies were diluted to 4 μg/ml in blocking solution and applied for 18 h at 4°C. A non-immune isotype IgG negative control (R&D Systems Inc., Minneapolis, MN, USA) diluted to a matching concentration as the primary antibody, was also included for each tissue. Antibody localisation was detected by sequential application of biotinylated horse anti-mouse IgG (Vector Laboratories, Burlingame, CA, USA) diluted 1:200 in blocking solution for 30 min and an avidin-biotin complex conjugated to HRP (Vectastain ABC Elite kit; Vector Laboratories, Burlingame, CA, USA). The substrate used was diaminobenzidine (DAB) (Zymed, San Francisco, USA) forming an insoluble brown precipitate. Sections were then counterstained in Harris hematoxylin (Sigma Diagnostics, St. Louis, USA). Sections from normal endometrium were used as positive controls and included in each immunostaining run to provide quality control.

### pSTAT3 and SOCS3 immunohistochemistry

Immunohistochemistry for pSTAT3 and SOCS3 was conducted using polyclonal rabbit anti-mouse (Cell Signalling Technology Inc., MA, USA) and monoclonal rabbit anti-human (Clone C204) (Immuno-Biological Laboratories Inc., MN, USA) antibodies respectively as previously shown [9], at final concentration of 0.09 μg/ml and 1 μg/ml respectively.

Formalin fixed sections were deparaffinized in histosol and rehydrated in a graded series of ethanol. Endogenous activity was blocked by incubation in 3% H_2_O_2 _in methanol for 10 min. Non-specific staining was blocked using blocking solutions consisting of 10% normal swine serum (in-house) and 2% normal human serum for pSTAT3 and 10% normal goat serum (Vector Laboratories) and 2% normal human serum for SOCS3, each diluted in 1×TBS for 30 min. Primary antibodies were diluted in the appropriate blocking solution and applied for 18 h at 4°C. A non-immune isotype IgG negative control (R&D Systems) diluted to a matching concentration as the primary antibody, was also included for each tissue. Antibody localisation was detected by sequential application of biotinylated swine anti rabbit IgG (DAKO, Glostrup, Denmark) or biotinylated goat anti-rabbit IgG (Vector Laboratories) diluted 1:200 in blocking solution correspondingly for 30 min and an avidin-biotin complex conjugated to HRP (Vectastain ABC Elite kit, Vector Laboratories). The substrate used was diaminobenzidine (DAB) (Zymed), which forms an insoluble brown precipitate. Sections were then counterstained in Harris hematoxylin (Sigma Diagnostics). Sections from normal pre-menopausal endometrium were used as positive controls and included in each immunostaining run to provide quality control.

### Endometrial epithelial cancer and non-cancer cell lines

The endometrial carcinoma cells ECC-1, HEC-1A and Ishikawa cells were cultured in DMEM/F12 (1:1), McCoy's 5A and DMEM (Invitrogen, Victoria, Australia) respectively supplemented with 10% fetal calf serum (SAFC Biosciences, Victoria, Australia), 1% L-glutamine (Sigma-Aldrich Pty. Ltd) and 1% antibiotic-antimycotic (Invitrogen, Victoria, Australia). The non-cancer human endometrial epithelial (HES) cell line [26] was obtained from Dr. Douglas Kniss (Ohio State University, Columbus, OH). Cells were maintained in RPMI 1640 (Sigma-Aldrich Pty. Ltd) supplemented with 10% FCS, 1% L-glutamine and 1% antibiotic- antimycotic. Confluent cells were transferred into serum free medium for 24 hours prior to treatment

### IL11 regulation of pSTAT3 and SOCS3 in human endometrial cancer cell lines

The endometrial cancer cell lines ECC-1, HEC-1A and Ishikawa and or HES cells were treated with diluents control, IL11 (1, 10, 100, 500 ng/ml) for 15 minutes or 4 hours. Phosphorylated STAT3 and total STAT3 abundance (15 min) and SOCS3 protein abundance (4 hours; treatment time was determined from previous studies in endometrial cells) were analysed by Western blot as previously described [9] and briefly as follows. Cells were grown to confluence, the medium aspirated and cells washed with ice-cold sterile PBS, twice on ice. Cells were lysed and scraped in ice-cold lysis buffer containing 50 mM Tris Base, 150 mM NaCl, 2 mM EDTA, 2 mM EGTA, 25 mM NaF, 25 mM β-glycerolphosphate, pH 7.5 and 2 μl/well protease inhibitors cocktail set III (AEBSF, HCl 100 mM, aprotinin 80 μM, bestatin 5 μM, E-64 1.5 mM, leupeptin hemisulfate 2 mM, pepstatin A 1 mM (Calbiochem, San Diego, CA, USA). Cell extracts were then centrifuged at 12000 rpm for 30 min at 4°C, and supernatant protein quantified using the BCA protein assay kit (Pierce, Rockford, IL, USA). Equal amounts of total protein were then resolved on SDS-PAGE gels and transferred to nitrocellulose membranes. All membranes were incubated with Ponceau S (Sigma) to ensure equal protein loading in all lanes. The membranes were blocked with 5% nonfat dry milk in Tris-buffered saline with 0.1% Tween (TBST) and probed separately with antibodies specific for phosphorylated STAT3 (Tyr705, Cell Signaling Technology Inc.) (1:1000), total STAT3 (Cell Signaling Technology), (1:1000) or SOCS3 (IBL Co. LTD, Gunma, Japan). The membranes were washed in TBST then incubated for 1 h with horseradish peroxidase (HRP)-conjugated rabbit secondary antibody (Dako Cytomation, Glostrup, Denmark) (1:1500). Finally, the HRP activity was detected using enhanced chemilluminescence reagent (Pierce, Rockford, IL, USA). To determine the specificity of IL-11 in the cells a specific IL11 antagonist was used (provided by Commonwealth serum Laboratories, Melbourne, Australia) [27].

All in vitro cell culture experiments were performed in two independent experiments in duplicate.

### Semiquantitative analysis of immunostaining and statistical analysis

Positive staining was scored semiquantitatively by two independent observers, blind to the identity of the tissue, with an intensity score assigned as 0 (negative) to 3 (maximal staining intensity). All statistical analyses were performed using GraphPad Prism. Data was analysed by the non-parametric Kruskal-Wallis test followed by the Dunn's post-hoc test. Differences were considered significant at P < 0.05.

## Results

### IL11 is upregulated in uterine fluid of women with endometrial cancer

**Figure 1:**
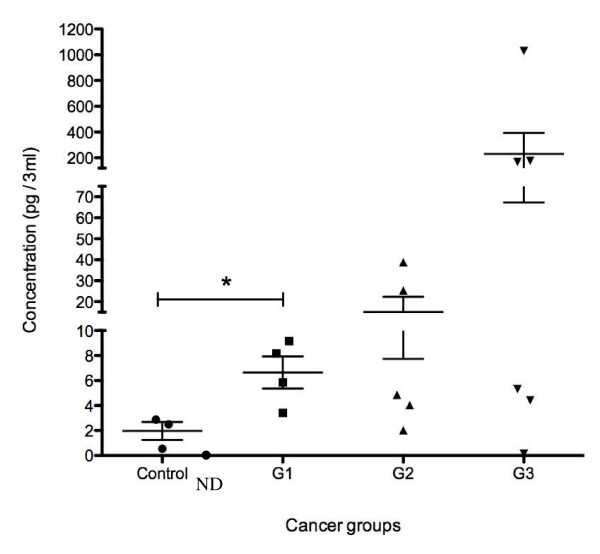
**IL11 is present in uterine washings in women with endometrial cancer**. Uterine washings were collected from women with Grade 1 (N = 4), Grade 2 (N = 5) and Grade 3 (N = 6) cancer and IL11 was measured by ELISA. Individual data shown for each parameter. Data represented as mean ± SEM. ND-not detectable * represents P < 0.05 compared to postmenopausal control.

IL11 was detectable in 3 of 4 postmenopausal controls and in all the flushings from the Grade 1-3 tumours (Fig 1). IL11 levels in uterine flushings from women with Grade 1 cancers were higher than that of postmenopausal control women (p < 0.05) (Fig 1). Uterine flushings from proliferative phase control women ranged from very low to undetectable IL11 and were therefore not included in the results and data analysis. Similarly, an additional three uterine washings from postmenopausal women were assayed for IL11 but had undetectable IL11 and were not included in the data analysis. Overall, IL11 levels in uterine flushings in the cancer patients were higher than the postmenopausal controls although this did not reach significance (Fig 1). There was a sub-group of women with Grade 3 cancers that had very high levels of IL11 in the uterine flushings (Fig 1).

### Immunolocalisation of IL11 and its specific receptor, IL11Rα in endometrial cancer and endometrium from post-menopausal women

**Figure 2:**
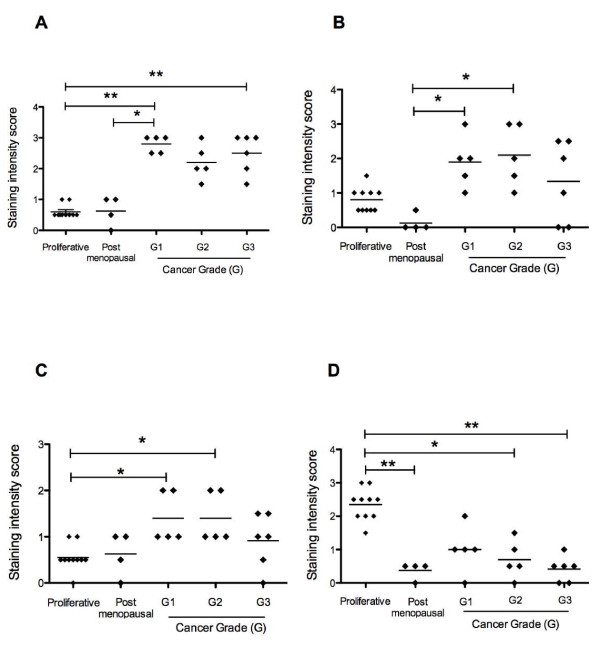
**Immunohistochemical localisation scores in endometrial cancer and postmenopausal endometrium**. Immunostaining of IL11 (A), IL11Rα (B), pSTAT3 (C) and SOCS3 (D) in tumor epithelial cells in endometrial carcinoma. Relative staining intensities are represented as 0 (no staining) to 3 (maximal staining). Post-menopausal women were controls (n = 4) while endometrial carcinomas were from Grades 1-3 (Grade 1: n = 5, Grade 2: n = 5, Grade 3: n = 6). Individual data shown for each parameter. Results presented as mean. Proliferative = proliferative phase of the menstrual cycle. * P < 0.05, ** P < 0.001 compared to postmenopausal control.

**Figure 3:**
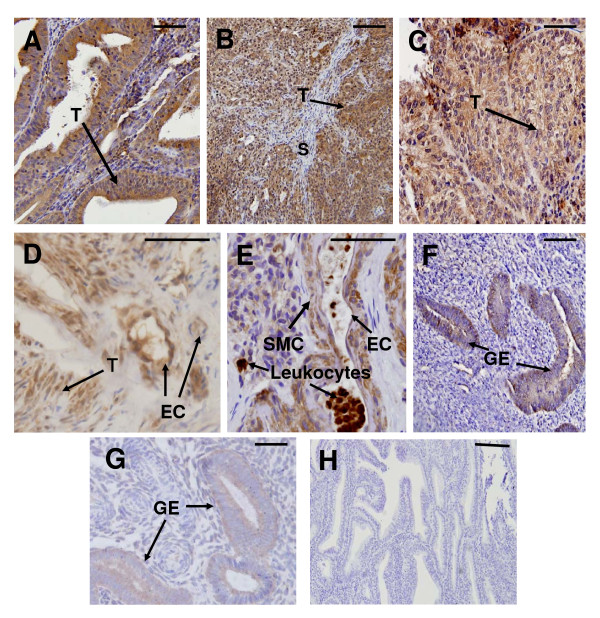
**IL11 immunolocalisation in endometrial cancer and postmenopausal endometrium**. Photomicrographs representing IL11 staining in endometrial cancers of (A) Tumour Grade 1, (B) Tumour Grade 2, (C-E) Tumour Grade 3, (F) postmenopausal endometrium, (G) secretory phase endometrium and (H) negative control. Positive staining shown as brown pigment with blue nuclear counterstain. Scale bars = 50 μm. (T) cancer epithelial cells, (GE) glandular epithelium, (EC) vascular endothelial cells, (SMC) smooth muscle cells and (S) stromal cells.

**Figure 4:**
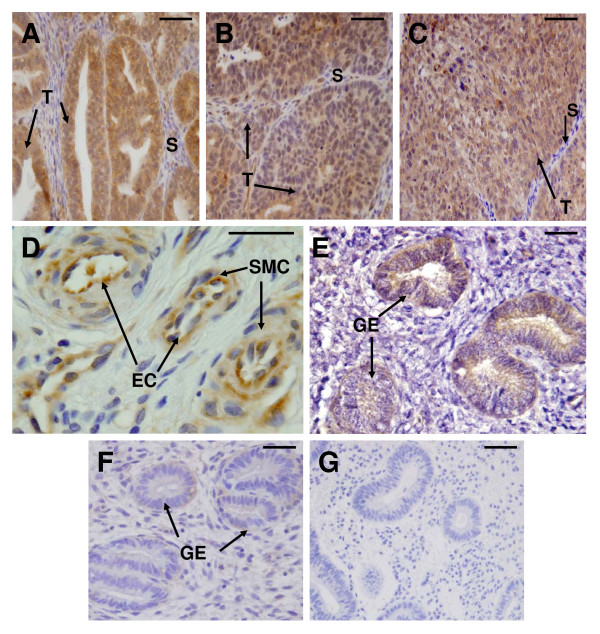
**IL11Rα immunolocalisation in endometrial cancer and postmenopausal endometrium**. Photomicrographs representing IL11Rα staining in endometrial cancer of (A) Tumour Grade 1, (B) Tumour Grade 2, (C-D) Tumour Grade 3, (E) postmenopausal endometrium, (F) secretory phase endometrium and (G) negative control. Positive staining shown as brown pigment with blue nuclear counterstain. Scale bars = 50 μm. (T) tumour epithelial cells, (GE) glandular epithelium, (EC) vascular endothelial cells, (SMC) smooth muscle cells and (S) stromal cells

Positive immunostaining for IL11 was detected in all cancer tissues examined; meanwhile IL11Rα staining was present in all cancer tissues except two from grade 3 (Fig 2A and 2B). In all tissues, IL11 and IL11Rα immunoreactivity was mainly localised to epithelial cells of tumour origin (Fig 3A-C and 4A-C respectively). Very little immunoreactive IL11 and IL11Rα was seen in the stromal compartment of the tumours (Fig 3A-C and 4A-C respectively). By contrast, only low levels of IL11 and IL11Rα staining was evident in epithelial cells while stromal cells were negative in endometrium from postmenopausal women and proliferative phase endometrium (Fig 2A and 2B, Figure 3F and 4E). IL11 immunostaining was significantly higher in epithelial tumour cells from Grades 1 and 3 but not Grade 2 tissue compared to endometrial epithelial cells from proliferative phase women (p < 0.05 in G1 and p < 0.001 in G3, Fig 1A) but was higher only in Grade 1 compared to postmenopausal women. There was no significant difference in IL11 staining in epithelial cancer cells between the tumour grades (Fig 2A). IL11Rα staining was higher in epithelial tumour cells in Grade 1 and 2 but not Grade 3 compared to endometrial epithelial cells from control postmenopausal women (p < 0.05, Fig 2B). Similar to IL11, there was no significant difference in IL11Rα staining in epithelial cancer cells between the tumour grades (Fig 2B).

IL11 staining was also present in vascular endothelial and smooth muscle cells in Grade 3 tumours (Fig 3D-E). Similarly, positive staining for IL11Rα was seen in vascular smooth muscle and endothelial cells in Grade 3 tumours but not in Grades 1 and 2 (Fig 4D). No staining for IL11 and IL11Rα was seen in vascular endothelial and smooth muscle cells in postmenopausal endometrium or proliferative phase endometrium (data not shown). Intense staining for IL11 was seen in subpopulations of leukocytes infiltrating the cancer glands in four of the six Grade 3 tumours (Fig 3E). Secretory phase endometrium served as positive controls for IL11 and IL11Rα and demonstrated positive IL11 and IL11Rα staining in glandular epithelium as previously reported (Fig 3G and Fig 4F) [12]. No immunostaining was detected in the IL11 and IL11Rα negative controls (Fig 3H and 4G).

### Immunolocalisation of pSTAT3 and SOCS3 in endometrial cancer tissue and endometrium from post-menopausal women

**Figure 5:**
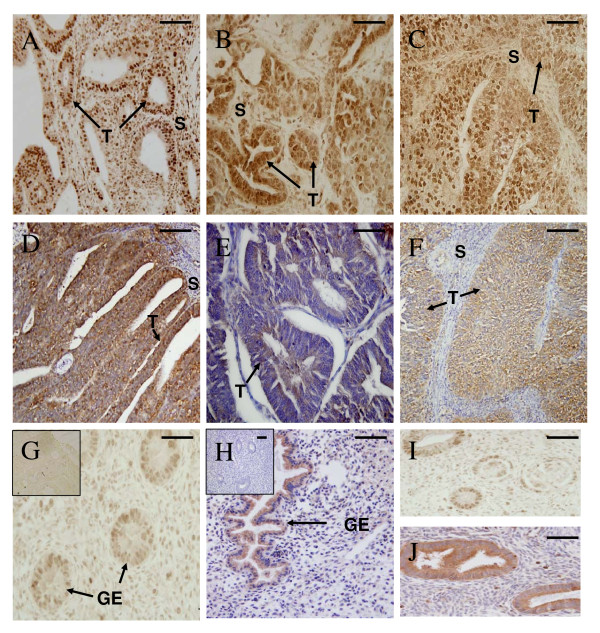
**pSTAT3 and SOCS3 localisation in endometrial cancer and postmenopausal endometrium**. Photomicrographs representing pSTAT3 (A-C) and SOCS3 (D-F) in endometrial cancers. A and D are Tumour Grade 1; B and E are Tumour Grade 2; C and F are Tumour Grade 3; G and H are control postmenopausal endometrium (G shows pSTAT3 and H shows SOCS3). I and J are positive controls for pSTAT3 and SOCS3 respectively showing secretory phase endometrium (insets are negative controls for each). Positive staining shown as brown pigment with blue nuclear counterstain. Negative controls (IgG isotype controls) are shown as insets for pSTAT (G) and SOCS3 (H). Scale bars = 50 μm. (T) tumour epithelial cells, (GE) glandular epithelium and (S) stromal cells

Staining for pSTAT3 was detected in epithelial and stromal compartments in the endometrial carcinomas and postmenopausal endometrium (Fig 5A-C and 5G). pSTAT3 immunostaining was low in the postmenopausal epithelium and proliferative phase. pSTAT3 was significantly increased in the cancer epithelium in Grades 1 and 2 compared to proliferative phase epithelium (Fig 2C). pSTAT3 staining in the tumour stroma was low to moderate but was minimal in the endometrium from postmenopaual women (Fig 5A-C and 5G). While there was an increase in pSTAT3 immunostaining intensity in the Grades 1 and 2 compared to postmenopausal epithelium, it did not reach significance (Fig 5C). There were no statistical differences in tumour stroma between cancer grades and also between each cancer grade and postmenopausal endometrium (data not shown). SOCS3 localised primarily to the endometrial cancer epithelium in all grades of carcinomas (Fig 5D-F). There was minimal staining for SOCS3 in endometrial epithelial cells from the postmenopausal women (Fig 5H). SOCS3 in proliferative phase epithelium was significantly higher compared to epithelium in post-menopausal controls and all Tumour Grades (Fig 2D). However, there were no significant difference in SOCS3 staining in the epithelial tumour cells between tumour grades (Fig 2D).

### IL11 regulation of pSTAT3 and SOCS3 in human endometrial cancer cell lines

Overall, all the human endometrial cancer cell lines (ECC-1: 5 pg/10^6 ^cells, HEC-1A: 3 pg/10^6 ^cells, Ishikawa cells: 6 pg/10^6 ^cells) and the endometrial epithelial cell line HES, secreted very low levels of IL11 under serum free conditions. The cells were subsequently cultured in serum free conditions to examine the effect of IL11 on pSTAT3/STAT3 and SOCS3 protein abundance.

**Figure 6:**
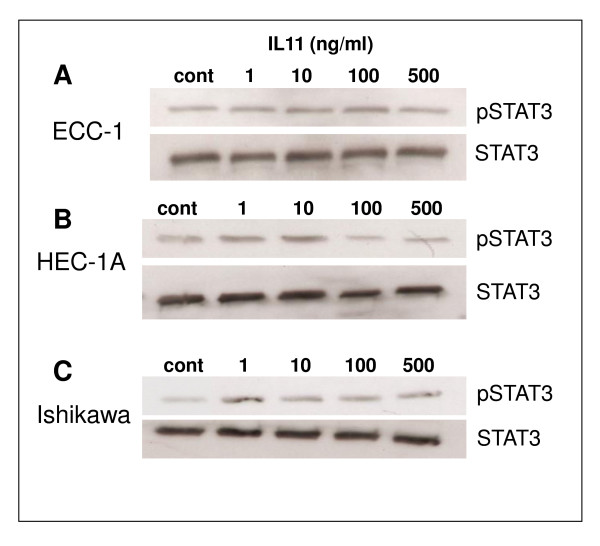
**Effect of IL-11 on STAT3 phosphorylation in human endometrial cancer cell lines**. Cells were cultured with IL-11 (1-500 ng/ml) for 15 min. A: ECC-1 cells. B: HEC-1A cells. C: Ishikawa cells. Cell lysates (30 μg protein) were electrophoresed by SDS-PAGE and immunoblotted with anti-p(Tyr705)STAT3 (top panel) or anti-STAT3 (bottom panel) followed by HRP-conjugated rabbit antiserum and visualized by chemiluminescence. cont = control

The effect of IL-11 on pSTAT3 and STAT3 in human endometrial epithelial cancer cell lines was examined by Western blot (Fig 6A top). Addition of IL-11 to ECC-1 cells weakly stimulated pSTAT3 at 100 pg/ml while there was no activation with all other concentrations. By contrast, IL11 stimulated pSTAT3 from 1-10 ng/ml in HEC-1A and 1 ng/ml in Ishikawa endometrial carcinoma cells respectively compared to diluent control treated cells (Fig 6B and 6C). STAT3 protein abundance was not affected at any IL11 concentration tested in all carcinoma cell lines (Fig. 6A).

**Figure 7:**
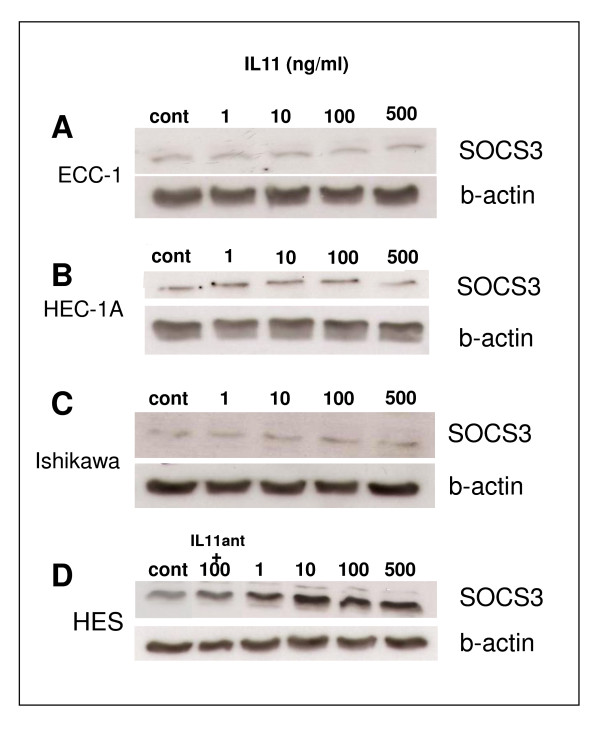
**Effect of IL-11 on SOCS3 in human endometrial cancer and non-cancer cell lines**. Cells were cultured with IL-11 (1-500 ng/ml) for 4 hours. A: ECC-1 cells. B: HEC-1A cells. C: Ishikawa cells. D: HES cells (non-cancer endometrial epithelial cell line). Cell lysates (30 μg protein) were electrophoresed by SDS-PAGE and immunoblotted with anti-SOCS3 (top panel) or anti-b-actin (loading control; bottom panel) followed by HRP-conjugated rabbit antiserum and visualized by chemiluminescence. cont = control IL11 ant = IL11 antagonist

To determine the effect of IL-11 on SOCS3 protein abundance, endometrial carcinoma and non-carcinoma (HES) cells were treated with IL-11 for 4 hours and SOCS3 abundance examined at 0 (before treatment) and 4 hrs as previously described [9]. SOCS3 protein abundance in ECC-1 cells did not change with addition of IL11 (Fig. 7A). In HEC-1A (Fig. 7B) and Ishikawa carcinoma cells (Fig. 7C), there was an upregulation of SOCS3 protein following the addition of 100 ng/ml IL11 compared to respective controls. In non-carcinoma HES cells, SOCS3 protein increased after addition of IL11 from 1-500 ng/ml (Fig. 7D). Addition of IL11 antagonist with 100 ng/ml IL11 reduced SOCS3 protein compared to controls (Fig. 7D).

## Discussion

This study was the first to show that IL11 protein was increased in uterine fluid and endometrial tumour epithelial cells in women with Grade 1 endometrial carcinoma compared to postmenopausal women. It further demonstrated that IL11's main endometrial signalling molecules, pSTAT3 and SOCS3, were produced by endometrial cancer cells. IL11 was shown to signal via pSTAT3 and SOCS3 in human endometrial cancer cell lines.

Endometrial glandular epithelial products are primarily secreted apically into the uterine lumen therefore we investigated the levels of IL11 in uterine flushings. In agreement with our study, a previous study has suggested that factors present in uterine washings may confirm the presence of endometrial cancer [23]. IL11 levels in uterine washings were very high (10-100 fold) in a cohort of women with Grade 3 cancers compared to the other tumour grades and controls. As endometrial cancer progresses, the epithelial cancer cells lose their polarity. Our study suggests that non-polarised endometrial cancer epithelial cells may also secrete products into the uterine lumen. It is also possible that IL11 may be secreted by the cancer associated leukocytes into the uterine lumen in the Grade 3 tumours thereby contributing to the IL11 levels found in the lavage fluid.

Previous studies have shown that in cycling endometrium, IL11 and IL11Rα predominantly localise to human endometrial glandular epithelium and decidualized human endometrial stromal cells [12,28]. Endometrial IL11 protein production alters with cyclical variation; in the glandular epithelium it is low in the proliferative phase of the menstrual cycle and increases in the mid-late secretory phase [12]. However, since endometrial cancer affects predominantly post-menopausal women, we compared the levels of IL11, IL11Rα, pSTAT3 and SOCS3 in endometrial cancer tissue to endometrial tissue from post-menopausal.

In agreement with our study, IL11 localised predominantly to cancer epithelial cells in a recent report [13]. IL11 mRNA was reported to be higher in endometrial cancer tissue compared to endometrial tissue from proliferative phase tissue, while differences in the level of IL11 protein between the groups was not reported [13]. Our data demonstrated that IL11 protein was significantly elevated specifically in endometrial epithelial tumor cells early in the Grade 1 tumours compared to postmenopausal controls reflecting the data in uterine washings. This suggests that IL11 levels in uterine washings may be useful as an endometrial cancer marker.

IL11Rα protein was upregulated in endometrial epithelial tumour cells compared to endometrial epithelium from postmenopausal women. Strong staining for both IL11 and IL11Rα was identified in tumour vascular endothelial and smooth muscle cells as recently reported [13].

IL11 localised to leukocytes only in the advanced Grade 3 tumours and not in control postmenopausal endometrium. Numerous studies report that tumour associated macrophages promote angiogenesis and correlate with poor prognosis [29]. In endometrial cancer, tumour associated macrophages are associated with vascular space invasion and myometrial invasion [30]. It is likely that factors produced by tumour associated leukocytes contribute to tumourigenesis.

In agreement with the present study, IL11 is significantly upregulated in several non-endometrial cancers. IL11 and IL11Rα transcript levels are linked to breast cancer prognosis - breast tumours with a poor prognostic index show a high level of IL11 [31]. Similarly, IL11 and IL11Rα protein are highly expressed in human colorectal adenocarcinoma and IL11Rα levels correlate with clinicopathological factors [32]. IL11 is also increased in gastric cancer [14]. Overall, these studies indicate that IL11 may play a role in tumour formation.

Tumour development and progression depends on cell adherence to extracellular matrix, proliferation, migration and invasion of tumour cells followed by their metastasis into other tissues and on escaping immune detection and destruction. Our previous studies show that IL11 increases the adhesion of human endometrial epithelial cells to various extracellular matrix molecules and to human trophoblast, at least in part by regulating adhesion molecule mRNA expression and protein production [10]. Endometrial extracellular matrix molecules appear to be targets of IL11 actions in mouse implantation sites [33]. IL11 also regulates the migration and invasion of human trophoblast, a process that is highly regulated but nevertheless has many similarities with tumour cell invasion [21,22]. Furthermore, IL11 and IL11Rα expression correlate with invasion and proliferation in human gastric and colorectal tumours [34,35].

It remains to be determined whether IL11 similarly regulates tumour cell adhesion, migration and invasion in endometrial cancers. Angiogenesis is also a key determinant of tumour formation [36] and hence the localization of IL11 and IL11Rα to vascular smooth muscle and endothelial cells in the present study suggest a potential role in angiogenesis. In the stomach, IL11 increases angiogenesis accelerating ulcer healing in mice [37].

IL11Rα protein is a proposed candidate target for both human osteosarcoma and also bone metastasis [16]. Furthermore IL11 alters the expression of proliferative and cytoprotective genes and promotes pre-tumorigenic cellular changes in mice in vivo suggesting that IL11 is involved early in tumourigenesis [14]. pSTAT3 staining intensity tended to be higher in the tumour epithelial cells compared to endometrium from postmenopausal women although it did not reach significance likely due to the large variability in staining intensity within the control group of women. By contrast, pSTAT3 intensity was higher in Grade 1 and 2 tumours compared to endometrial glandular epithelium from proliferative phase tissue. This suggests that caution must be used when comparing endometrial cancer proteins with proliferative phase endometrium.

Overall, SOCS3 imunostaining intensity was low in epithelium from postmenopausal women and all tissues from the cancer patients. There was higher SOCS3 staining in endometrial glandular epithelium from proliferative phase endometrium compared to all other groups. This suggests that SOCS3 has different functions in cycling endometrium compared to endometrium from postmenopausal women and endometrial cancer.

IL11 increases pSTAT3 and SOCS3 protein in differentiating human endometrial stromal cells [38]. STAT3 which is phosphorylated by numerous cytokines, growth factors and oncogenetic proteins, is constitutively phosphorylated in many human cancer tissues and cell lines [18]. STAT3 target genes are implicated in multiple steps of tumour metastasis including cell invasion, survival, renewal and angiogenesis and thus pSTAT3 can be regarded as a pivotal regulator of tumour metastasis [18]. It was of interest in the present study to investigate whether specifically IL11 regulated pSTAT3 and SOCS3 in cancer cells as both have been shown to be involved in numerous tumours. The intense staining identified for pSTAT3 in endometrial cancer associated epithelium and stroma, suggests a role in both stromal and epithelial compartments for pSTAT3 in endometrial tumour formation. IL11 is predominantly restricted to cancer epithelium and not cancer associated stromal fibroblasts, suggesting that in the cancer stroma, factors other than IL11 regulate pSTAT3. Whether IL11 alone activates STAT3 phosphorylation in endometrial cancer cells remains to be elucidated.

Several studies have shown that SOCS proteins including SOCS3 are expressed in tumours including head and neck cancer [39,40], gastric carcinoma [41], chronic myeloid leukemia [42], melanoma [43] and prostate cancer [44,45]. SOCS3 is upregulated in prostate cancer and inhibits the induction of apoptosis by cAMP [44].

In the present study SOCS3 staining intensity was absent or very minimal in tumour epithelial cells in the Grade 3 cancer specimens perhaps similarly indicating a reduced sensitivity to SOCS3 in endometrial cancers although this remains to be determined. In normal breast epithelial cells SOCS3 is induced, while in several breast cancer cell lines SOCS3 is weakly activated. In breast tumour cells, it has been postulated that the IFNγ induced anti-proliferative effects are reduced due to a lower sensitivity to SOCS3 induction [19]. Our in vitro studies identified that IL11 (from 1 ng/ml) stimulated SOCS3 protein abundance in non-carcinoma HES cells. By contrast IL11 weakly stimulated SOCS3 protein at 100 ng/ml in the carcinoma HEC-1A and Ishikawa cells possibly suggesting reduced sensitivity in endometrial cancer cells. The mechanisms by which this may occur are unknown. The consequences of this reduced sensitivity could be that IL11 signalling is unregulated in endometrial cancer cells. This however this remains to be determined and the functional significance remains to be elucidated.

## Conclusions

Our study suggests that IL11 in uterine washings may be useful as an early marker of endometrial cancer. It is also the first study to demonstrate that IL11 protein is upregulated in Grade 1 endometrial cancers compared to postmenopausal endometrial epithelium and suggests that IL11 signalling is active in endometrial cancer cells. The present study suggests that IL11, along with its specific receptor and downstream signalling molecules pSTAT3 and SOCS3, are likely to play a complex role in the progression of endometrial carcinoma. Functional studies are required to elucidate the role of IL11 in tumourigenesis and determine its potential as a prognostic marker and therapeutic target for endometrial cancer. Large scale studies are required to determine whether IL11 in uterine washings may be useful as a diagnostic marker for endometrial cancer.

## Competing interests

The authors declare that they have no competing interests.

## Authors' contributions

JY performed immunohistochemistry, quantitative ELISA, western blots, data analysis and assisted in drafting the manuscript. LAS contributed to tissue grading and collection of biopsies. TJ co-ordinated patient recruitment, tissue cancer grading and collected the biopsies. PKN provided technical assistance with the immunohistochemistry. ED conceived of the study, designed and co-ordinated the study, participated in data analysis and interpretation and drafted the manuscript. All authors read and approved the manuscript.
